# Ganoderma lucidum spore extract improves sleep disturbances in a rat model of sporadic Alzheimer’s disease

**DOI:** 10.3389/fphar.2024.1390294

**Published:** 2024-04-24

**Authors:** Yu Qin, Yan Zhao, Xiao Hu, Xi Chen, Yan-Ping Jiang, Xue-Jun Jin, Gao Li, Zhen-Hao Li, Ji-Hong Yang, Guo-Liang Zhang, Su-Ying Cui, Yong-He Zhang

**Affiliations:** ^1^ Department of Pharmacology, School of Basic Medical Science, Peking University, Beijing, China; ^2^ Key Laboratory of Natural Medicines of the Changbai Mountain, Ministry of Education, College of Pharmacy, Yanbian University, Yanji, China; ^3^ Department of Pharmacy, Yanbian University Hospital, Yanji, China; ^4^ Zhejiang ShouXianGu Pharmaceutical Co., Ltd., Wuyi, Zhejiang, China

**Keywords:** *G. lucidum* spores, sleep disorder, sporadic Alzheimer’s disease, γ-aminobutyric acid, neuroinflammation

## Abstract

**Introduction:** *Ganoderma lucidum* (*G. lucidum, Lingzhi*) has long been listed as a premium tonic that can be used to improve restlessness, insomnia, and forgetfulness. We previously reported that a rat model of sporadic Alzheimer’s disease (sAD) that was induced by an intracerebroventricular injection of streptozotocin (ICV-STZ) showed significant learning and cognitive deficits and sleep disturbances. Treatment with a *G. lucidum* spore extract with the sporoderm removed (RGLS) prevented learning and memory impairments in sAD model rats.

**Method:** The present study was conducted to further elucidate the preventive action of RGLS on sleep disturbances in sAD rats by EEG analysis, immunofluorescence staining, HPLC-MS/MS and Western blot.

**Results:** Treatment with 720 mg/kg RGLS for 14 days significantly improved the reduction of total sleep time, rapid eye movement (REM) sleep time, and non-REM sleep time in sAD rats. The novelty recognition experiment further confirmed that RGLS prevented cognitive impairments in sAD rats. We also found that RGLS inhibited the nuclear factor-κB (NF-κB)/Nod-like receptor family pyrin domain-containing 3 (NLRP3) inflammatory pathway in the medial prefrontal cortex (mPFC) in sAD rats and ameliorated the lower activity of γ-aminobutyric acid (GABA)-ergic neurons in the parabrachial nucleus (PBN).

**Discussion:** These results suggest that inhibiting the neuroinflammatory response in the mPFC may be a mechanism by which RGLS improves cognitive impairment. Additionally, improvements in PBN-GABAergic activity and the suppression of neuroinflammation in the mPFC in sAD rats might be a critical pathway to explain the preventive effects of RGLS on sleep disturbances in sAD.

## 1 Introduction

*Ganoderma lucidum* (*G. lucidum*) is a medicinal and edible traditional Chinese medicine that has been used for hundreds of years as a supplement to increase life span and enhance a healthier life (Cui and Zhang, 2019; Ren et al., 2022). Over 400 bioactive compounds, including triterpenoids, polysaccharides, steroids, fatty acids, and nucleotides, have been identified from the fruit body, mycelia, and spores of *G. lucidum*, which are the main sources of its pharmacological and therapeutic properties, such as antiaging, antitumor, antiulcer, antiviral, antibacterial, sleep-promoting, antioxidant, anticancer, antiinflammatory, antidiabetic, cardioprotective, hypolipidemic, and immunomodulatory effects (Swallah et al., 2023). Sporoderm-removed *G. lucidum* spore (RGLS) is a deeply processed product with the advantage of yielding higher levels bioactive components and improving absorption (Soccol et al., 2016). As previously reported, high-resolution mass spectrometry showed that RGLS had a high triterpenoids content, which was much higher than a *G. lucidum* spore powder extract without wall breaking (Li et al., 2020). Only a few peaks were detected in the extract of sporangium powder that did not break the wall, suggesting that intact spores act as a barrier to the release of endospore components (Li et al., 2020). Studies have shown that compared to wall-broken *G. lucidum* spore powder, RGLS can significantly improve the levels of active constituents (polysaccharides and triterpenoids), and thereby enhance the antitumor activity (Shi et al., 2021). Currently, RGLS has been shown to attenuate atherosclerosis and aortic calcification (Zheng et al., 2023), inhibit the proliferation, migration and invasion of esophageal squamous cell carcinoma (Liu and Zeng, 2021), and enhance the sensitivity of ovarian cancer to cisplatin (Cen et al., 2022). Our previous study showed that RGLS application prevented memory impairments and the reduction of neurotrophic factors in the hippocampus in a rat model of sporadic Alzheimer’s disease (sAD) that was induced by an intracerebroventricular injection of streptozotocin (ICV-STZ) (Zhao et al., 2021).

Alzheimer’s disease is a neurodegenerative brain disease that is mainly characterized by memory loss, cognitive decline, and pathological signs of extracellular neuritic plaques that are composed of amyloid-β (Aβ) protein and intracellular neurofibrillary tangles that contain phosphorylated Tau protein (Scheltens et al., 2016). These AD-like pathologies may be initiated by neuroinflammation (Leng and Edison, 2021). Additionally, AD patients often experience sleep disturbances (Ju et al., 2014). The association between AD and sleep disorders is well known to have a bidirectional relationship, which was linked by neuroinflammation (Irwin and Vitiello, 2019). Sleep disorders are generally considered a consequence of AD, which can be explained by progressive neurodegenerative damage in sleep-regulating brain regions. However, sleep disturbances are also suggested to be a candidate risk factor of AD because it leads to neuroinflammation and the pathological accumulation of Aβ and Tau protein (Guo et al., 2020). Theoretically, therapeutic interventions for sleep enhancement could alleviate this vicious bidirectional relationship and consequently prevent the development of AD. However, clinical studies have shown considerable uncertainty about the balance of benefits and side effects of classic hypnotics, including benzodiazepines, sedative antidepressants, and melatonin (McCleery and Sharpley, 2020; Blackman et al., 2021). Therefore, a better understanding of the mechanisms that underlie the association between sleep disturbances and AD has the potential to improve therapeutic interventions for AD.

*G. lucidum* spores have strong antineuroinflammatory effects and have been conventionally applied as a sedative agent (Cui et al., 2012). However, few studies have evaluated RGLS for the treatment of sleep disturbances in an animal model of sAD. Interestingly, ICV-STZ rats exhibit memory impairments and sleep disturbances that are similar to AD patients (Cui et al., 2018), with a notable increase in inflammation in discrete brain regions, including the prefrontal cortex (PFC) and hippocampus (Souza et al., 2022). These findings suggest that neuroinflammation might be associated with sleep disturbances and cognitive impairment in ICV-STZ rats. In the present study, we explored therapeutic effects of RGLS on cognitive impairment and sleep disturbances in ICV-STZ rats and sought to reveal the underlying mechanisms of the protective effects of RGLS in sAD rats.

## 2 Materials and methods

### 2.1 Animals

Adult male Sprague-Dawley rats (250–280 g) were obtained from the Animal Center of Peking University (Beijing, China). All rats were individually housed in plastic cages with *ad libitum* access to food and water at a temperature of 25°C ± 2°C and 55%–65% humidity. An artificial 12 h/12 h light/dark cycle (lights on 9:00 a.m.) was regulated in the animal house. All rats were given 1 week to acclimate to the new environment before surgery. All experimental procedures were conducted according to the “Reporting animal research: Explanation and elaboration for the ARRIVE guidelines 2.0” (Percie du Sert et al., 2020) and approved by the Peking University Committee on Animal Care and Use (permission no. LA 2020279).

### 2.2 Electroencephalography/electromyography and cannula surgery

According to previously described methods (Cui et al., 2018), under isoflurane (5% induction and 2% maintenance) anesthesia, the rats were implanted with electrodes for the polysomnographic recording of electroencephalograms (EEGs) and electromyograms (EMGs) and implanted with guide cannulas (O.D. 0.64 mm × I.D. 0.25 mm, C.C. 1.2 mm, RWD Life science Co., Ltd., Shenzhen, China) for drug administration into the lateral ventricle. After positioning the rats in a stereotaxic instrument, the guide cannula was implanted for the injection of STZ or vehicle (anterior/posterior, −0.8 mm; medial/lateral, −1.5 mm; dorsal/ventral, −3.5 mm). The cannulas and EEG electrodes were then fixed to the skull with dental cement, anchored by four stainless-steel screws. Two stainless-steel wire electrodes were placed in the dorsal neck muscles for EMG recordings. All electrodes were attached to a miniature connector. After surgery, the rats were placed on a heated pad, observed for at least 30 min, and then returned to their home cage. After surgical implantation, the rats were injected with penicillin for 3 days and allowed to recover for 7 days before the experiments. For habituation, the animals were connected to the recording apparatus at least 1 day before the experiments.

### 2.3 Drug treatment and experimental design

Streptozotocin (catalog no. S0130, Sigma-Aldrich, St. Louis, MO, United States) was administered at a dose of 3 mg/kg in 4 μL artificial cerebrospinal fluid (aCSF; 3525/25 mL, Tocris Bioscience, Bristol, UK). Streptozotocin was slowly and continually injected in the ventricle at a rate of 0.8 μL/min using a Hamilton microsyringe that was attached to a 33-gauge injection cannula (Plastics One, Roanoke, VA, United States) over a 5 min period. The needle was left in place for an additional 3 min to allow for diffusion.

RGLS was provided by Zhejiang ShouXianGu Pharmaceutical Co. Ltd. (batch no. 20181101, Zhejiang, China). This product was approved by the State Food and Drug Administration of China in 2016 (China health food approval no. G20160280). Representative chromatograms of RGLS were published in a previous report (Zhao et al., 2021). A voucher specimen (SXG-GLS-20181006016) was deposited in our laboratory and at the company. The samples were kept in the Department of Pharmacology, School of Basic Medical Science, Peking University. For oral administration, RGLS was dissolved in distilled water to a final concentration of 45, 90, and 180 mg/mL. The rats were weighed daily before administration and the administration volume was calculated based on body weight (4 mL/kg). Dose of RGLS of 180, 360, and 720 mg/kg were used in this study.

**FIGURE 1:**
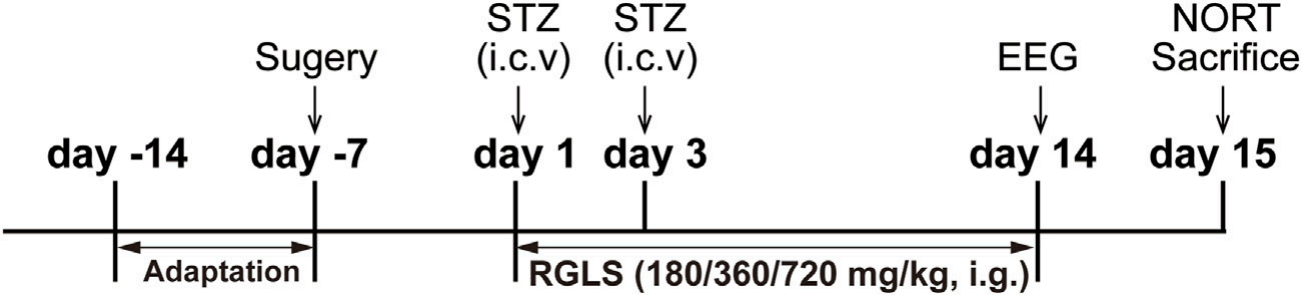
Experimental design.

**FIGURE 2:**
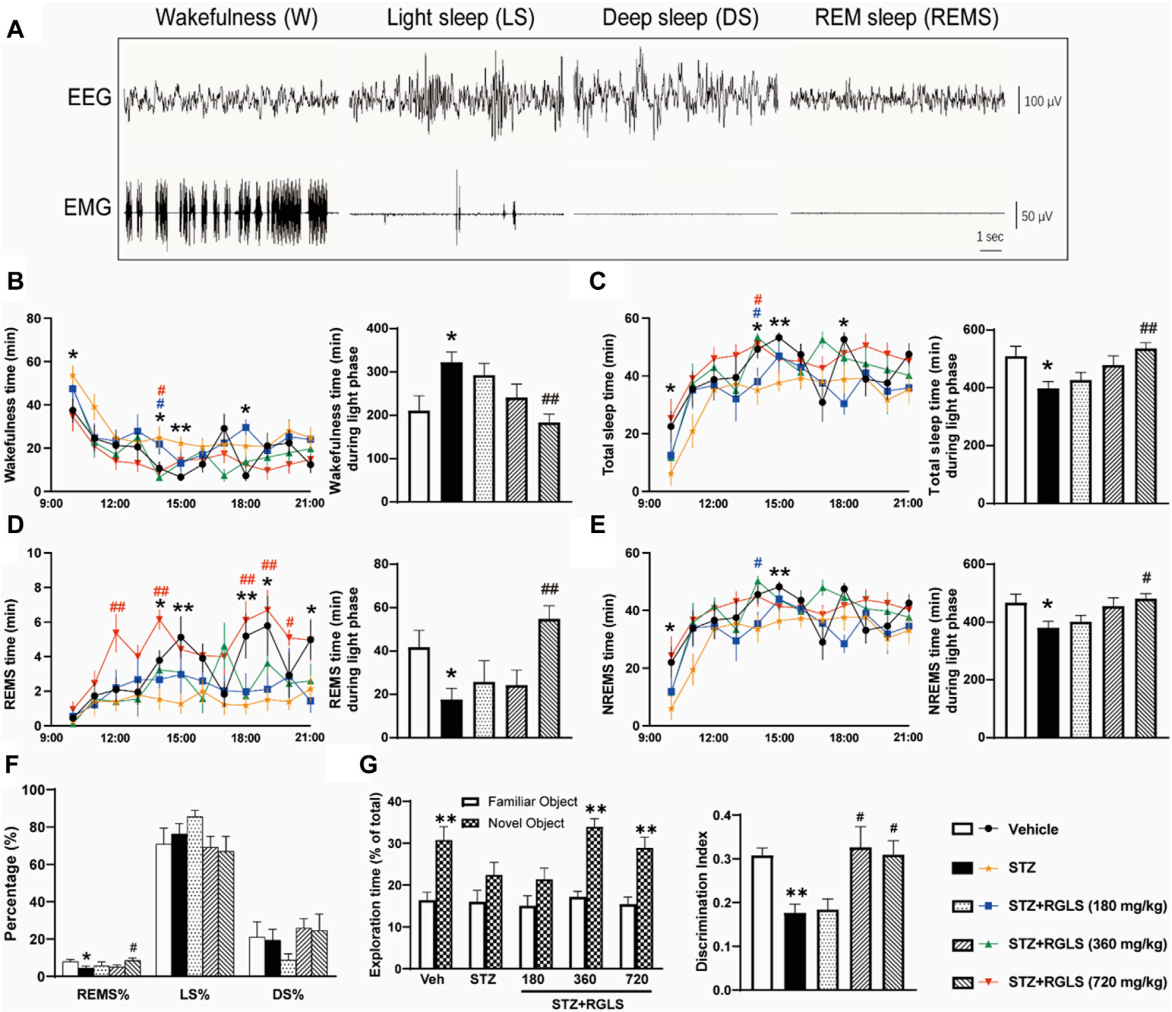
RGLS ameliorates sleep-wake cycle disruption in ICV-STZ rats. **(A)** The representative EEG spectrograms and EMG traces. **(B–E)** Time spent in wakefulness, total sleep, rapid eye movement sleep (REMS) and non-REM sleep (NREMS) across 12 h during light phase. **(F)** Percentage of REMS, light sleep (LS) and deep sleep (DS) time relative to total sleep (REMS%, LS%, DS%). **(G)** Exploration time and the discrimination ratio in the NORT. All data are presented as means ± SEM (*n* = 7–9). Compared to vehicle group, *p < 0.05, **p < 0.01; Compared to ICV-STZ group, #*p* < 0.05, ##*p* < 0.01.

As shown in Figure 1, the rats were randomly assigned to five groups: vehicle, STZ, STZ + RGLS (180 mg/kg), STZ + RGLS (360 mg/kg), and STZ + RGLS (720 mg/kg). After 7 days of recovery from surgery, the STZ and STZ +RGLS groups received two intracerebroventricular injections of STZ at an interval of 48 h. RGLS treatment occurred once daily at 9:00 a.m. from day 1 to day 14. The vehicle group was treated with aCSF intracerebroventricularly and distilled water intragastrically. Subsequently, EEG and EMG recordings were conducted on day 14 after RGLS treatment. As nocturnal animals, rats are activity during night time. Therefore, the novel object recognition test (NORT) were performed from 00:00 to 03:00 a.m. on day 15. In addition, 1 h-block sleep/wake-stage analysis showed that the differences across vehicle, STZ and RGLS groups were most significant from 13:00 to 15:00 p.m. on day 14 (Figure 2), so the rats were sacrificed at 14:00 p.m. on day 15. Brains were rapidly removed to a prechilled brain matrix with a 1.0 mm coronal slice thickness (RWD Life Technology, Shenzhen, Guangzhou, China; catalog no. 68709). Several brain regions that are involved in sleep-wake regulation, including the ventrolateral preoptic nucleus (VLPO), parabrachial nucleus (PBN), parafacial zone (PZ), and medial PFC (mPFC), were rapidly dissected according to the Paxinos and Watson rat brain atlas using a brain blade and frozen at −80°C for subsequent biochemical analysis (Paxinos and Watson, 1986).

### 2.4 EEG and EMG recordings and analysis

Sleep-wake stages were analyzed by EEG and EMG recordings for 12 h, from 9:00 a.m. to 9:00 p.m., on day 14 after RGLS administration. A lightweight shielded cable was plugged into the connector on the rat’s head and attached to a counterbalanced swivel that allowed free movement of the animal. All rats were studied in an electrically shielded box and noise-attenuated environment that was free from interruptions. Before sleep recording, the rats were connected to the recording apparatus for at least 1 day for habituation. The signals were routed to an electroencephalograph (Model MP 150; BIOPAC Systems, Goleta, CA, United States). The rats’ behaviors were monitored by video surveillance with EEG/EMG recordings. The signals were amplified, filtered (EEG, 0.5–30 Hz; EMG, 10–100 Hz), digitized at a sampling rate of 128 Hz, and recorded using AcqKnowledge software (BIOPAC Systems). The EEG and EMG recordings were analyzed in 10 s epochs using standard criteria using SleepSign 2.0 software (Kissei Comtec, Nagano, Japan). As shown in Figure 2A, sleep-wake states were automatically classified as wakefulness (W), rapid eye movement sleep (REMS), deep sleep (DS), and light sleep (LS). As a final step, the defined sleep-wake stages were manually examined and corrected based on the following criteria: wakefulness (low-amplitude EEG activity and high-voltage EMG activity), REMS (fast Fourier transform [FFT] theta percentage of EEG ≥60%, desynchronized EEG, the absence of tonic EMG, and occasional body twitches while maintaining a recumbent sleep posture), DS (FFT delta percentage of EEG ≥70%, large-amplitude, synchronous EEG with sleep spindles present, greatly diminished tonic EMG, eyes closed, and recumbent posture), and LS (FFT delta percentage of EEG <70%, high-amplitude slow or spindle EEG activity, and low-amplitude EMG activity). Non-rapid-eye-movement sleep (NREMS) time was equal to DS time + LS time. The total sleep (TS) time was equal to NREMS time + REMS time.

### 2.5 Novel object recognition test

The NORT is a validated and widely used test for assessing declarative memory and object recognition (Lueptow, 2017). The test was performed in a Plexiglas box (40 cm × 40 cm × 65 cm) with a white light on the top. The procedure consists of three phases: habituation, training, and testing. The rats were habituated individually to the testing box for 20 min in the habituation phase (24 h before the training phase). During the training phase, two identical objects were placed on opposite walls of the testing box, and the animals were allowed to freely explore the box for 10 min. The rats were then returned to their home cages and 2 h later placed again in the testing box in the presence of one of the familiar objects and one novel object for 10 min (testing phase). The rats’ behavior in each trial was video recorded. The exploration time was scored by an observer who was blind to the experimental conditions. Exploration was defined as sniffing, biting, licking, or touching the object with the nose. Turning around or sitting on the object was not considered exploratory behavior. During the test session, the discrimination index (DI) was calculated using the formula (B − A)/(B + A), where B is the time spent exploring the novel object and A is the time spent exploring the familiar object.

### 2.6 Estimation of GABA concentrations

Concentrations of γ-aminobutyric acid (GABA) were measured using high-performance liquid chromatography–dual mass spectrometry (HPLC–MS/MS). Briefly, tissue was transferred to a new tube and then mixed with 90 μL of a prechilled (4°C) methanol (0.1% formic acid)-aqueous (8:2, v/v) mixture and 10 μL of internal standard (IS; 2-chloro-L-phenylalanine [2-Cl-Phe]; 1 μg/mL). The mixture was homogenized using an ultrasonic homogenizer, followed by centrifugation at 20,000 × *g* for 20 min at 4°C. After centrifugation, the separated supernatant was transferred to a 2 mL autosampler, and 10 μL was injected into the system at a flow rate of 0.4 mL/min. The standard curve was prepared using the same procedure as the brain sample. Each data point is from an individual rat.

The HPLC–MS/MS system consisted of a Dionex UltiMate 3000 Ultra-HPLC system (Thermo, San Jose, CA, United States) and an API 4000Q Trap mass spectrometer (AB SCIEX, Foster City, United States) equipped with an electrospray ionization source interface. The optimized mass spectrometric parameters were set as the following: curtain gas, 15 psi; collision gas, 2; ion spray voltage, 5500 V for positive mode or −4500 V for negative mode; ion source temperature, 600°C; ion source gas 1, 55 psi; ion source gas 2, 55 psi. Accurate quantification was operated in multiple reaction monitoring mode. The transitions were m/z 104.1→87.1 (positive). The declustering potential was set at 35, and the collision energy (CE) was set at 16 V.

Chromatographic separation was performed on an Ultimate XB-C18 column (100 mm × 2.1 mm, 5 μm, Welch Materials). Mobile phase A consisted of water that contained 0.1% formic acid, and mobile phase B was acetonitrile. The temperature of the autosampler was set at 4°C. Gradient separation was set as the following: 0–1 min, 5% B; 1–3 min, 5%–60% B; 3.1–5 min, 5% B for column equilibration. The analysis was performed in a total run time of 5 min. Under these conditions, the retention time was 0.8 min. The above data were recorded and analyzed using AB SCIEX Analyst 1.6 software.

GABA was purchased from Sigma-Aldrich (catalog no. A2129, St. Louis, MO, United States). 2-Cl-Phe (98% purity; catalog no. C105993, Aladdin, Shanghai, China) was used as an IS. HPLC-grade acetonitrile and methanol were obtained from Fisher Chemical (Fisher Scientific, Shanghai, China).

### 2.7 Immunofluorescence staining and image analysis

Under chloral hydrate (300 mg/kg, i.p.) anesthesia, the rats were slowly perfused with 200 mL of 0.01 M phosphate-buffered saline (PBS), followed by 200 mL of 4% paraformaldehyde in PBS (pH 7.4). Whole brains were immediately removed, soaked in 4% paraformaldehyde at 4°C for 24 h, and then successively transferred to 20% sucrose and 30% sucrose at 4°C until the tissues sank. The brains were rapidly frozen in liquid optimal cutting temperature compound (catalog no. 4583, Sakura Finetek, CA, United States) that was cooled with a mixture of solid carbon dioxide and ethanol. Serial coronal brain sections were cut into 20 μm thickness on a cryostat microtome (Leica Microsystems UK, catalog no. CM 1950, Milton Keynes, UK) and stored at −20°C in cryoprotectant solution (48% PBS, 30% ethylene glycol, 20% glycerol, and 2% dimethylsulfoxide). Double immunofluorescence was performed as described previously with slight modifications (Cui et al., 2018). Briefly, the sections were washed in 0.01 M PBS (3 × 5 min). Antigen retrieval was then conducted in a metal bath at 96°C that contained 0.01 M citrate buffer (pH 6.0) for 5 min. After the sections were returned to room temperature naturally, slides were washed three times and permeabilized with 0.01 M PBS that contained 0.1% Triton X-100 for 20 min. After blocking with 10% nonspecific donkey serum for 40 min at room temperature, the slides were incubated in appropriate primary antibodies for glutamate decarboxylase 65 (GAD 65, 1:50, catalog no. 5843, Cell Signaling Technology, MA, United States) and c-Fos (1:100, catalog no. ab208942, Abcam, Cambridge, UK) diluted in 0.01 M PBS that contained 1.5% nonspecific donkey serum and 0.3% Triton X-100 for 48 h at 4°C. The sections were washed three times in 0.01 M PBS and incubated for 2 h at room temperature with appropriate secondary antibodies. Alexa Fluor 448-conjugated donkey anti-rabbit antibody (1:200, catalog no. AS035, ABclonal, Wuhan, China) and Alexa Fluor 594-conjugated goat anti-mouse antibody (1:150, catalog no. AS054, ABclonal, Wuhan, China) were used as secondary antibodies. Finally, nuclei were stained with DAPI.

Single images from the PBN for each rat were examined under a Leica TCS-SP8 STED 3X laser scanning confocal microscope (Leica, Germany). For quantitative analysis, Fiji software was used to measure integrated density. We quantified the results of immunofluorescence by counting GAD 65^+^ neurons and c-Fos ^+^GAD 65^+^ neurons per unit field of view.

### 2.8 Western blot

Tissues were homogenized in RIPA lysis buffer and complete protease and phosphatase inhibitors. The homogenate was centrifuged at 12,000 × *g* for 15 min at 4°C. Next, 5× loading buffer was added to the supernatant and then used to detect protein levels. The protein was separated by 10% sodium dodecyl sulfate-polyacrylamide gel electrophoresis and transferred to polyvinylidene difluoride membranes (Bio-Rad, Hercules, CA, United States). Membranes were blocked with 5% skim milk for 1 h at room temperature and incubated with primary antibodies in TBST buffer (Tris-buffered saline and 10.1% Tween-20) at 4°C overnight. The primary antibodies were anti-β-actin (1:2000, catalog no. AC026, Cell Signaling Technology, Danvers, MA, United States), anti-GAD 65 (1:1000, catalog no. 5843, Cell Signaling Technology, MA, United States), anti-phospho-NF-κB (p-NFκB, 1:1000, catalog no. AP1294, ABclonal, Wuhan, China), anti-NF-κB (1:1000, catalog no. A19653, ABclonal, Wuhan, China), anti-Nod-like receptor family pyrin domain-containing 3 (NLRP3; 1:1000, catalog no. ab263899, Abcam, Cambridge, UK), anti-caspase-1 (1:1000, catalog no. ab179515, Abcam, Cambridge, UK), and anti-apoptosis-associated speck-like protein (ASC; 1:500, catalog no. ab180799, Abcam, Cambridge, UK). After 3 × 5 min TBST washes, the blots were incubated with horseradish peroxidase-conjugated secondary antibodies (1:2000; Cell Signaling Technology, Danvers, MA, United States) for 2 h at room temperature and then washed with TBST buffer for 3 × 5 min. Lastly, protein expression was detected with the ECL Enhanced Kit (catalog no. RM00021, ABclonal, Wuhan, China) and subsequently analyzed densitometrically using ImageJ software. The results were normalized to the protein expression level of β-actin in each sample.

### 2.9 Data and statistical analysis

The data were analyzed using GraphPad Prism 8.0.1 software and are expressed as the mean ± SEM. The sample size was the number of independent values, and the statistical analysis was performed using these independent values. Data from the vehicle groups and ICV-STZ groups were compared using *t*-tests. Data from the ICV-STZ groups and RGLS-treated STZ groups were compared using one-way analysis of variance (ANOVA) followed by Tukey’s multiple-comparison *post hoc* test. Values of *p* < 0.05 were considered statistically significant.

## 3 Results

### 3.1 RGLS ameliorates sleep-wake cycle disruption in ICV-STZ rats

ICV-STZ is an animal model that is commonly used to simulate sAD (Grieb, 2016). Our previous study found that ICV-STZ rats also exhibited sleep disturbances 14 days after the STZ injection (Cui et al., 2018). Consistent with the previous study, ICV-STZ rats exhibited a significant increase in wakefulness (W, t _(14)_ = 2.754) and a significant decrease in the time spent in the sleep state (TS, t _(14)_ = 2.734) compared with vehicle rats during the light phase (*p* < 0.05, Figures 2B, C). Wakefulness and TS time were analyzed in 1 h blocks across 12 h. Differences were mainly observed in 9:00–10:00, 13:00–15:00, and 17:00–18:00 when ICV-STZ rats spent more time awake and less time asleep than vehicle rats. The daily administration of RGLS (720 mg/kg) that started on the day after the STZ injection prevented ICV-STZ-induced sleep deficits, reflected by a decrease in W time (F_(3, 26)_ = 6.381) and an increase in TS time (F_(3, 26)_ = 6.314). Additionally, RGLS (180 and 720 mg/kg) significantly decreased W time and increased TS time in 13:00–14:00. REMS time was significantly lower in ICV-STZ rats than in vehicle rats specifically in 13:00–15:00, 15:00–17:00, and 20:00–21:00 (t _(14)_ = 2.697, Figure 2D). Compared with ICV-STZ rats, RGLS (720 mg/kg) significantly increased REMS time and the time spent in REMS per 1 h in 11:00–12:00, 13:00–14:00, and 17:00–20:00 (F_(3, 26)_ = 6.52, *p* < 0.05 or *p* < 0.01). NREMS time was also significantly lower in ICV-STZ rats than in vehicle rats specifically in 9:00–10:00 and 14:00–15:00 (t _(14)_ = 2. 402, Figure 2E). RGLS (720 mg/kg) significantly increased NREMS time (F_(3, 26)_ = 4.403), and RGLS (180 mg/kg) significantly increased the time spent in NREMS per 1 h in 13:00–14:00. RGLS (720 mg/kg) also significantly reversed ICV-STZ-induced disturbances of sleep structure, reflected by normalization of the lower proportion of REMS relative to TS (*p* < 0.05, Figure 2F).

Furthermore, ICV-STZ rats showed a significant decrease in exploration time for the novel object and the DI in the NORT compared with vehicle rats (*p* < 0.01, Figure 2G), suggesting that the rats exhibited cognitive impairments 14 days after the STZ injection. The daily administration of RGLS (360 or 720 mg/kg) that started on the day after the STZ injection prevented ICV-STZ-induced cognitive impairments, reflected by an increase in exploration time for the novel object and the DI in the NORT (F_(3, 26)_ = 5.962). These results showed that RGLS prevented memory loss and cognitive decline in the sAD model in rats.

### 3.2 RGLS ameliorates cognitive deficits in ICV-STZ rats by inhibiting the NF-κB/NLRP3 pathway in the mPFC

Neuroinflammation is a key mechanism in the pathogenesis of neurodegenerative diseases that are associated with sleep disturbances (Irwin and Vitiello, 2019). ICV-STZ caused significant neuroinflammation in discrete brain regions that are involved in cognition and sleep regulation, and antiinflammatory treatment might reverse these behavioral disorders (Kumar et al., 2015).

**FIGURE 3:**
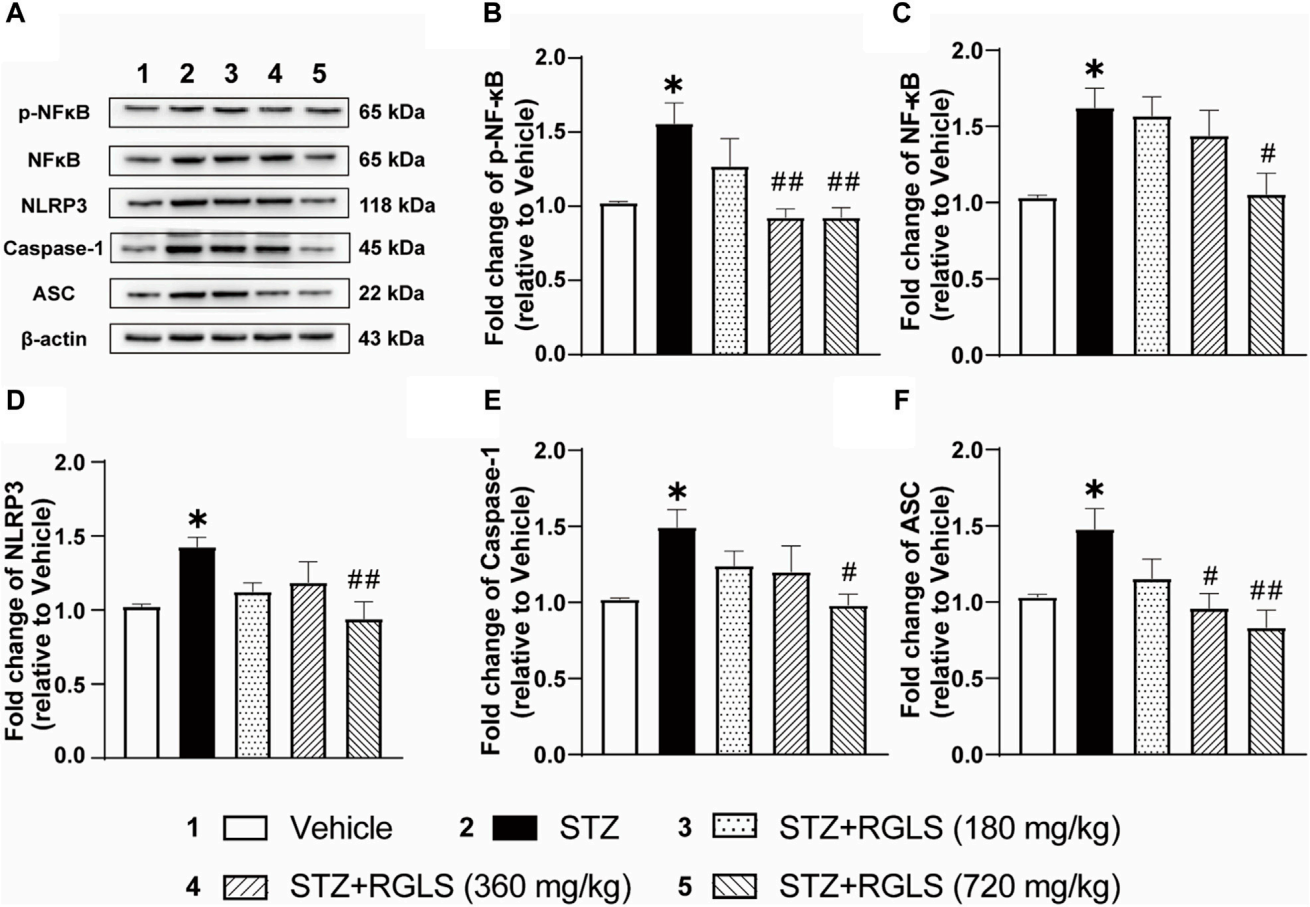
RGLS ameliorates neuroinflammation via inhibiting NF-κB/NLRP3 pathway in the mPFC in ICV-STZ rats. **(A)** Representative western blots showing the expression of p-NF-κB, NF-κB, NLRP3, Caspase-1 and ASC in the mPFC. **(B–F)** Quantitative analysis of the proteins for p-NF-κB, NF-κB, NLRP3, Caspase-1 and ASC in the mPFC. All data are presented as means ± SEM (*n* = 6). Compared to vehicle group, *p < 0.05, **p < 0.01; Compared to ICV-STZ group, #*p* < 0.05, ##*p* < 0.01.

A wide range of stimulations that appear during tissue damage resulting in NLRP3 activation and formation of an inflammasome complex. The NLRP3 inflammasome is a multimeric protein complex that consists of the danger sensor protein NLRP3, the signal adaptor protein ASC, and the cysteine protease caspase-1. Assembly and activation of the NLRP3 inflammasome is mediated by the NF-κB signaling pathway. In the present study, NF-κB/NLRP3 inflammatory signaling cascades were detected in the mPFC to explore the mechanism that underlies protective effects of RGLS in ICV-STZ rats. p-NF-κB, NF-κB, NLRP3, caspase-1, and ASC expression in the mPFC significantly increased in ICV-STZ rats compared with the vehicle group (p-NF-κB, t _(14)_ = 3.897; NF-κB, t _(14)_ = 4.654; NLRP3, t _(14)_ = 6.264; caspase-1, t _(14)_ = 4.104; ASC, t _(14)_ = 3.283, *p* < 0.01, Figures 3A–F). Once-daily RGLS treatment (360 and 720 mg/kg) completely reversed ICV-STZ-induced increases in p-NF-κB and ASC expression (p-NF-κB, F_(3, 28)_ = 6.153; ASC, F_(3, 28)_ = 5.595, *p* < 0.05 or *p* < 0.01, Figures 3B, F). RGLS treatment at 720 mg/kg reversed ICV-STZ-induced increases in NF-κB, NLRP3, and caspase-1 (NF-κB, F_(3, 28)_ = 3.403; NLRP3, F_(3, 28)_ = 4.094; caspase-1, F_(3, 28)_ = 3.114, *p* < 0.05 or *p* < 0.01, Figures 3C–E). These results indicated that RGLS suppressed neuroinflammation by inhibiting the NF-κB/NLRP3 pathway in the mPFC.

### 3.3 RGLS restored the deficit of GABAergic neurons in the PBN in ICV-STZ rats

**FIGURE 4:**
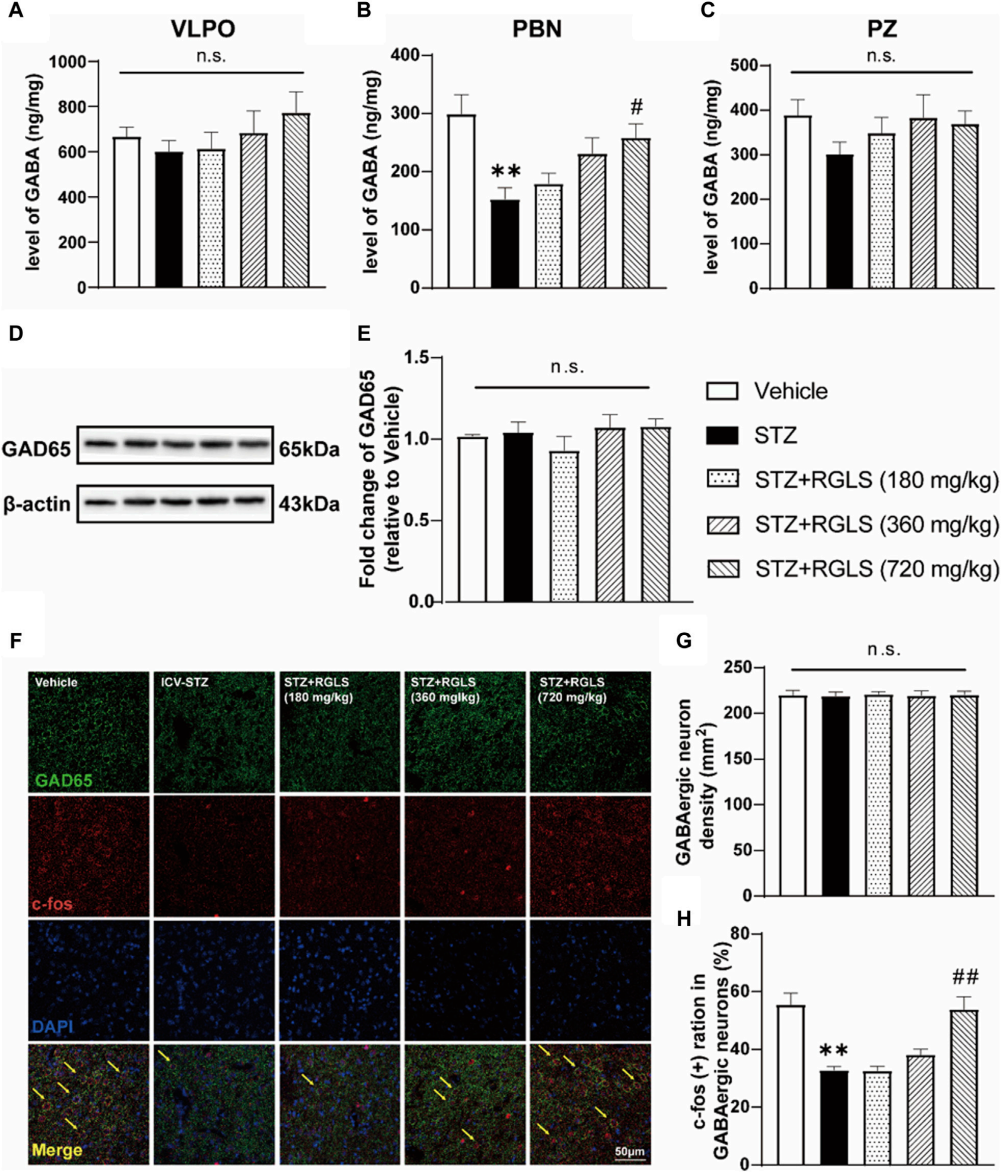
RGLS restored the deficit of GABAergic neurons in the PBN of ICV-STZ rats. **(A–C)** Levels of GABA in the VLPO, PBN and PZ. **(D)** Representative western blots showing the expression of GAD 65 in the PBN. **(E)** Quantitative analysis of the proteins for GAD 65 in the PBN. **(F)** Double-stained GAD 65 and c-Fos in the PBN. Yellow arrows indicate dual GAD 65^+^ and c-Fos^+^ neurons. **(G, H)** The density of GABA neurons and double-stained cells was calculated. All data are presented as means ± SEM (*n* = 6). Compared to vehicle group, *p < 0.05, **p < 0.01; Compared to ICV-STZ group, #*p* < 0.05, ##*p* < 0.01.

Sleep disturbances in AD often overlap with neuropathology in multiple sleep-wake-regulating regions of the brain (Lew et al., 2021). Similarly, the extensive dysfunction of sleep-promoting neurons has been observed in ICV-STZ rats (Cui et al., 2018). To investigate possible mechanisms of sleep-promoting effects of RGLS in ICV-STZ rats, we quantified GABA levels in the VLPO, PBN, and PZ using HPLC–MS/MS. The results showed that no significant changes in GABA levels in the VLPO or PZ in ICV-STZ rats, and RGLS did not affect GABA levels in these two regions (*p* > 0.05, Figures 4A, C). However, ICV-STZ resulted in a reduction of GABA in the PBN (t _(10)_ = 3.824, *p* < 0.01), whereas GABA levels significantly increased in the STZ + RGLS (720 mg/kg) group compared with the ICV-STZ group (F_(3, 20)_ = 4.697, *p* < 0.05; Figure 4B).

Previous research indicated that dysfunction in GABAergic signaling in the PBN might be associated with sleep disorders of sAD model rats. Intra-PBN injection of GABA_A_ receptor antagonist induced sleep disorders similar to those induced by ICV-STZ in rats, and intra-PBN injection of GABA improved sleep disorders in ICV-STZ rats. To further validate the role of the PBN in sleep-promoting effects of RGLS in ICV-STZ rats, we detected GAD 65, an enzyme that mediates synaptic GABA synthesis, by Western blot and quantified the density and activity of GABAergic neurons in the PBN using double-stained immunofluorescence. GAD 65 expression was unaffected by ICV-STZ or RGLS treatment (*p* > 0.05, Figures 4D, E). The density of GAD 65^+^ (GABAergic) neurons in the PBN was unaffected by the STZ injection and RGLS treatment (*p* > 0.05, Figures 4F, G). These results indicated that the alterations in GABA levels in the PBN might not be due to GAD 65 expression level. c-Fos expression is often considered an index of neuronal activation. The ratio of c-Fos^+^ GABAergic neurons in the PBN was lower in ICV-STZ rats than in vehicle rats (t _(10)_ = 5.497, *p* < 0.01, Figure 4H). RGLS treatment (720 mg/kg) completely reversed ICV-STZ-induced decreases in c-Fos expression in GABAergic neurons (F _(3, 20)_ = 14.96, *p* < 0.01). These results indicate that the sleep-promoting effects of RGLS in ICV-STZ rats might be related to the normalization of GABAergic neuronal activity in the PBN that was suppressed by STZ injections.

## 4 Discussion

*G. lucidum* has been used medicinally in Asia for more than 2000 years, and its extracts have exhibited sedative and memory-enhancing effects (Bishop et al., 2015). In recent years, pharmacological effects of *G. lucidum* on preventing and treating neurodegenerative diseases and its possible mechanisms of action have attracted much attention (Qin et al., 2017; Yu et al., 2020). RGLS is a deep-processed *G. lucidum* product with higher levels of polysaccharides and triterpenoids than its unbroken spore powder, and it is easier to be absorbed by the body (Soccol et al., 2016). The present study, together with our previous report (Zhao et al., 2021), showed that RGLS treatment improved memory deficits and attenuated sleep disturbances in ICV-STZ rats (Figure 2). We presume that the sleep-restoring effect of RGLS treatment may be beneficial for AD prevention in ICV-STZ rats.

Alzheimer’s disease patients often experience sleep disturbances (Ju et al., 2014). Sleep disturbances generally appear in the preclinical phase of AD and may serve as a predictor of neurodegeneration and cognitive dysfunction (Borges et al., 2019). One biologically plausible mechanism that links sleep disorders and AD is neuroinflammation. Briefly, sleep disturbances lead to abnormal microglial activation in the brain, which in turn contributes to dysregulatory neuroinflammatory responses and causes neuronal synapse loss, a reduction of Aβ clearance, Tau hyperphosphorylation, and neurotoxicity via multiple inflammatory cascades, all of which are thought to occur during the progression of AD dementia (Irwin and Vitiello, 2019; Si et al., 2023). A wide range of stimulations that appear during tissue damage, resulting in NLRP3 activation and formation of an inflammasome complex and finally induces the secretion of activated cytokines and results inflammatory responses. NLRP3 inflammasome signaling is a characteristic of sleep disorders and AD pathophysiology (Milner et al., 2021; Amini et al., 2022). Studies have shown that sleep deprivation activates the NLRP3 inflammasome and causes neuroinflammation in the rat brain (Smith et al., 2021). Furthermore, recent reports posit that the NLRP3 inflammasome is essential for Aβ and Tau pathology and leads to cognitive decline in mice (Heneka et al., 2013; Ising et al., 2019). The present study showed that ICV-STZ dramatically activated the NF-κB/NLRP3 inflammatory pathway in the mPFC (Figure 3). This neuroinflammation might be associated with sleep impairments and cause cognitive dysfunction in ICV-STZ rats. We also found that RGLS treatment suppressed ICV-STZ-induced neuroinflammation, reflected by NF-κB and NLRP3 complex proteins returning to normal levels in the mPFC. The antiinflammatory effect of RGLS might be related to its sleep-restoring effect. Reciprocally, the sleep-restoring effects of RGLS might also be a consequence of antiinflammation because the daytime sleep-wake cycle in ICV-STZ rats was shown to be restored by minocycline (Vicente et al., 2023). Nevertheless, both sleep-restoring and antineuroinflammatory effects of RGLS may alleviate the deleterious bidirectional relationship between sleep disturbances and AD and thus be beneficial for AD prevention. In previous studies, we found that RGLS prevented the formation of learning and memory impairments in the same rat model of sAD by decreasing Aβ expression and Tau hyperphosphorylation and modulating BDNF-TrkB signaling in the hippocampus (Zhao et al., 2021). These findings suggest that both hippocampal and mPFC mechanisms are involved in the development of learning, memory, cognitive, and sleep disorders in sAD.

Sleep disorders in AD patients are mainly characterized by a longer sleep latency, night-time wakefulness, and daytime napping (Ju et al., 2014). In terms of sleep architecture, AD patients have a shorter duration of REMS than healthy individuals of the same age, and NREMS is also reduced (Borges et al., 2019). The present study found that ICV-STZ rats exhibited an increase in wakefulness and decreases in NREMS and REMS during the light period (Figure 2), sleep patterns that are similar to AD patients. RGLS alleviated sleep disturbances in the present ICV-STZ rat model of sAD, accompanied by the normalization in GABAergic neuronal activity in the PBN (Figures 2, 4). *G. lucidum* extracts were shown to potentiate pentobarbital-induced sleep in rats and have moderate affinity for GABA_A_ receptors, suggesting that GABAergic-dependent mechanisms are involved in hypnotic effects of *G. lucidum* (Cho et al., 2010). Sleep disturbances in AD often begin early in disease pathogenesis when abnormal Tau protein begins to accumulate in multiple sleep/wake-regulating regions of the brainstem, such as the PBN (Rub et al., 2016). The PBN is located in the dorsolateral pons and consists mainly of glutamatergic and GABAergic neurons (Fulwiler and Saper, 1984). The PBN plays a critical role in arousal through glutamatergic signaling (Fuller et al., 2011). Glutamatergic signaling receives GABAergic inhibitory inputs from sleep-promoting regions to facilitate the consolidation of NREMS, whereas a group of GABAergic neurons in the PBN are active during NREMS and are involved in controlling the NREMS state (Torterolo et al., 2011; Qiu et al., 2016). Additionally, REMS deprivation induces excitation in the PBN (Clement et al., 2011). These reports suggest that the PBN plays an important role in sleep-wake regulation. In the present study, we found that c-Fos expression decreased in GABAergic neurons in the PBN in ICV-STZ rats, and GABA levels also significantly decreased in the PBN in ICV-STZ rats. These results suggest that the lower activity of GABAergic signaling in the PBN might be responsible for the promotion of wakefulness and suppression of NREMS and REMS in ICV-STZ rats. RGLS treatment reversed these abnormalities in GABAergic signaling in the PBN and alleviated sleep disturbances in ICV-STZ rats, suggesting that the therapeutic benefits of RGLS for AD prevention may involve normalization of the function of sleep/wake-regulating brain regions.

## 5 Conclusion

The present findings, together with our previous work (Zhao et al., 2021), provide new insights into the protective effect of RGLS in sAD rats. RGLS can reduce the expression of Aβ in the hippocampus, reduce Tau hyperphosphorylation, modulate BDNF-TrkB signaling, inhibit the neuroinflammatory NF-κB/NLRP3 pathway in the mPFC, and improve GABAergic system function in the PBN. These mechanisms may be closely related to the prevention and improvement of learning deficits, cognitive dysfunction, and sleep disturbances in sAD rats. These findings suggest that RGLS may be a potential therapeutic agent for the prevention and treatment of cognitive impairments and sleep disturbances in sAD.

## Data Availability

The raw data supporting the conclusion of this article will be made available by the authors, without undue reservation.

## Abbreviations

*G. lucidum*, *Ganoderma lucidum*; RGLS, removed the sporoderm of a *G. lucidum* spore extract; sAD, sporadic Alzheimer’s disease; ICV-STZ, intracerebroventricular injected with streptozotocin; Aβ, beta-amyloid; EEG, electroencephalogram; EMG, electromyogram; NORT, novel object recognition test; VLPO, ventrolateral preoptic nucleus; PBN, parabrachial nucleus; PZ, parafacial zone; mPFC, medial prefrontal cortex; GABA, γ-aminobutyric acid; p-NFκB, phospho-NF-kB; NF-κB, nuclear factor kappa B; NLRP3, NOD-like receptor containing pyrin-domain 3; ASC, apoptosis-associated speck-like protein.

## References

[B1] Amini M. Yousefi Z. Ghafori S. S. Hassanzadeh G. (2022). Sleep deprivation and NLRP3 inflammasome: is there a causal relationship? Front. Neurosci. 16, 1018628. 10.3389/fnins.2022.1018628 36620464 PMC9815451

[B2] Bishop K. S. Kao C. H. Xu Y. Glucina M. P. Paterson R. R. Ferguson L. R. (2015). From 2000years of Ganoderma lucidum to recent developments in nutraceuticals. Phytochemistry 114, 56–65. 10.1016/j.phytochem.2015.02.015 25794896

[B3] Blackman J. Swirski M. Clynes J. Harding S. Leng Y. Coulthard E. (2021). Pharmacological and non-pharmacological interventions to enhance sleep in mild cognitive impairment and mild Alzheimer's disease: a systematic review. J. Sleep. Res. 30 (4), e13229. 10.1111/jsr.13229 33289311 PMC8365694

[B4] Borges C. R. Poyares D. Piovezan R. Nitrini R. Brucki S. (2019). Alzheimer's disease and sleep disturbances: a review. Arq. Neuropsiquiatr. 77 (11), 815–824. 10.1590/0004-282X20190149 31826138

[B5] Cen K. Chen M. He M. Li Z. Song Y. Liu P. (2022). Sporoderm-broken spores of Ganoderma lucidum sensitizes ovarian cancer to cisplatin by ROS/ERK signaling and attenuates chemotherapy-related toxicity. Front. Pharmacol. 13, 826716. 10.3389/fphar.2022.826716 35264959 PMC8900012

[B6] Cho S. M. Shimizu M. Lee C. J. Han D. S. Jung C. K. Jo J. H. (2010). Hypnotic effects and binding studies for GABA(A) and 5-HT(2C) receptors of traditional medicinal plants used in Asia for insomnia. J. Ethnopharmacol. 132 (1), 225–232. 10.1016/j.jep.2010.08.009 20804838

[B7] Clement O. Sapin E. Berod A. Fort P. Luppi P. H. (2011). Evidence that neurons of the sublaterodorsal tegmental nucleus triggering paradoxical (REM) sleep are glutamatergic. Sleep 34 (4), 419–423. 10.1093/sleep/34.4.419 21461384 PMC3064553

[B8] Cui S. Y. Song J. Z. Cui X. Y. Hu X. Ma Y. N. Shi Y. T. (2018). Intracerebroventricular streptozotocin-induced Alzheimer's disease-like sleep disorders in rats: role of the GABAergic system in the parabrachial complex. CNS Neurosci. Ther. 24 (12), 1241–1252. 10.1111/cns.13032 30014576 PMC6490136

[B9] Cui X. Zhang Y. (2019). Neuropharmacological effect and clinical applications of Ganoderma (Lingzhi). Adv. Exp. Med. Biol. 1182, 143–157. 10.1007/978-981-32-9421-9_5 31777017

[B10] Cui X. Y. Cui S. Y. Zhang J. Wang Z. J. Yu B. Sheng Z. F. (2012). Extract of Ganoderma lucidum prolongs sleep time in rats. J. Ethnopharmacol. 139 (3), 796–800. 10.1016/j.jep.2011.12.020 22207209

[B11] Fuller P. M. Sherman D. Pedersen N. P. Saper C. B. Lu J. (2011). Reassessment of the structural basis of the ascending arousal system. J. Comp. Neurol. 519 (5), 933–956. 10.1002/cne.22559 21280045 PMC3119596

[B12] Fulwiler C. E. Saper C. B. (1984). Subnuclear organization of the efferent connections of the parabrachial nucleus in the rat. Brain Res. 319 (3), 229–259. 10.1016/0165-0173(84)90012-2 6478256

[B13] Grieb P. (2016). Intracerebroventricular streptozotocin injections as a model of Alzheimer's disease: in search of a relevant mechanism. Mol. Neurobiol. 53 (3), 1741–1752. 10.1007/s12035-015-9132-3 25744568 PMC4789228

[B14] Guo T. Zhang D. Zeng Y. Huang T. Y. Xu H. Zhao Y. (2020). Molecular and cellular mechanisms underlying the pathogenesis of Alzheimer's disease. Mol. Neurodegener. 15 (1), 40. 10.1186/s13024-020-00391-7 32677986 PMC7364557

[B15] Heneka M. T. Kummer M. P. Stutz A. Delekate A. Schwartz S. Vieira-Saecker A. (2013). NLRP3 is activated in Alzheimer's disease and contributes to pathology in APP/PS1 mice. Nature 493 (7434), 674–678. 10.1038/nature11729 23254930 PMC3812809

[B16] Irwin M. R. Vitiello M. V. (2019). Implications of sleep disturbance and inflammation for Alzheimer's disease dementia. Lancet Neurol. 18 (3), 296–306. 10.1016/S1474-4422(18)30450-2 30661858

[B17] Ising C. Venegas C. Zhang S. Scheiblich H. Schmidt S. V. Vieira-Saecker A. (2019). NLRP3 inflammasome activation drives tau pathology. Nature 575 (7784), 669–673. 10.1038/s41586-019-1769-z 31748742 PMC7324015

[B18] Ju Y. E. Lucey B. P. Holtzman D. M. (2014). Sleep and Alzheimer disease pathology--a bidirectional relationship. Nat. Rev. Neurol. 10 (2), 115–119. 10.1038/nrneurol.2013.269 24366271 PMC3979317

[B19] Kumar A. Sharma S. Prashar A. Deshmukh R. (2015). Effect of licofelone--a dual COX/5-LOX inhibitor in intracerebroventricular streptozotocin-induced behavioral and biochemical abnormalities in rats. J. Mol. Neurosci. 55 (3), 749–759. 10.1007/s12031-014-0414-4 25204299

[B20] Leng F. Edison P. (2021). Neuroinflammation and microglial activation in Alzheimer disease: where do we go from here? Nat. Rev. Neurol. 17 (3), 157–172. 10.1038/s41582-020-00435-y 33318676

[B21] Lew C. H. Petersen C. Neylan T. C. Grinberg L. T. (2021). Tau-driven degeneration of sleep- and wake-regulating neurons in Alzheimer's disease. Sleep. Med. Rev. 60, 101541. 10.1016/j.smrv.2021.101541 34500400 PMC8862638

[B22] Li Z. Shi Y. Zhang X. Xu J. Wang H. Zhao L. (2020). Screening immunoactive compounds of Ganoderma lucidum spores by mass spectrometry molecular networking combined with *in vivo* zebrafish assays. Front. Pharmacol. 11, 287. 10.3389/fphar.2020.00287 32256359 PMC7093641

[B23] Liu G. Zeng T. (2021). Sporoderm-removed Ganoderma lucidum spore powder may suppress the proliferation, migration, and invasion of esophageal squamous cell carcinoma cells through PI3K/AKT/mTOR and erk pathway. Integr. Cancer Ther. 20, 15347354211062157. 10.1177/15347354211062157 34841952 PMC8649442

[B24] Lueptow L. M. (2017). Novel object recognition test for the investigation of learning and memory in mice. J. Vis. Exp. 126, 55718. 10.3791/55718 PMC5614391 28892027

[B25] McCleery J. Sharpley A. L. (2020). Pharmacotherapies for sleep disturbances in dementia. Cochrane Database Syst. Rev. 11 (11), CD009178. 10.1002/14651858.CD009178.pub3 33189083 PMC8094738

[B26] Milner M. T. Maddugoda M. Gotz J. Burgener S. S. Schroder K. (2021). The NLRP3 inflammasome triggers sterile neuroinflammation and Alzheimer's disease. Curr. Opin. Immunol. 68, 116–124. 10.1016/j.coi.2020.10.011 33181351

[B27] Paxinos G. Watson C. (1986). The rat brain in stereotaxic coordinates. Netherlands: Elsevier, 6.

[B28] Percie du Sert N. Ahluwalia A. Alam S. Avey M. T. Baker M. Browne W. J. (2020). Reporting animal research: Explanation and elaboration for the ARRIVE guidelines 2.0. PLoS Biol. 18 (7), e3000411. 10.1371/journal.pbio.3000411 32663221 PMC7360025

[B29] Qin C. Wu S. Q. Chen B. S. Wu X. X. Qu K. Y. Liu J. M. (2017). Pathological changes in APP/PS-1 transgenic mouse models of Alzheimer's disease treated with Ganoderma lucidum preparation. Zhongguo Yi Xue Ke Xue Yuan Xue Bao 39 (4), 552–561. 10.3881/j.issn.1000-503X.2017.04.015 28877835

[B30] Qiu M. H. Chen M. C. Fuller P. M. Lu J. (2016). Stimulation of the pontine parabrachial nucleus promotes wakefulness via extra-thalamic forebrain circuit nodes. Curr. Biol. 26 (17), 2301–2312. 10.1016/j.cub.2016.07.054 27546576 PMC5025760

[B31] Ren Z. Ding H. Zhou M. Chan P. (2022). Ganoderma lucidum modulates inflammatory responses following 1-methyl-4-phenyl-1,2,3,6-tetrahydropyridine (MPTP) administration in mice. Nutrients 14 (18), 3872. 10.3390/nu14183872 36145248 PMC9505693

[B32] Rub U. Stratmann K. Heinsen H. Turco D. D. Seidel K. Dunnen W. (2016). The brainstem tau cytoskeletal pathology of Alzheimer's disease: a brief historical overview and description of its anatomical distribution pattern, evolutional features, pathogenetic and clinical relevance. Curr. Alzheimer Res. 13 (10), 1178–1197. 10.2174/1567205013666160606100509 27264543

[B33] Scheltens P. Blennow K. Breteler M. M. de Strooper B. Frisoni G. B. Salloway S. (2016). Alzheimer's disease. Lancet 388 (10043), 505–517. 10.1016/S0140-6736(15)01124-1 26921134

[B34] Shi Y. J. Zheng H. X. Hong Z. P. Wang H. B. Wang Y. Li M. Y. (2021). Antitumor effects of different Ganoderma lucidum spore powder in cell- and zebrafish-based bioassays. J. Integr. Med. 19 (2), 177–184. 10.1016/j.joim.2021.01.004 33495135

[B35] Si Z. Z. Zou C. J. Mei X. Li X. F. Luo H. Shen Y. (2023). Targeting neuroinflammation in Alzheimer's disease: from mechanisms to clinical applications. Neural Regen. Res. 18 (4), 708–715. 10.4103/1673-5374.353484 36204826 PMC9700083

[B36] Smith C. Trageser K. J. Wu H. Herman F. J. Iqbal U. H. Sebastian-Valverde M. (2021). Anxiolytic effects of NLRP3 inflammasome inhibition in a model of chronic sleep deprivation. Transl. Psychiatry 11 (1), 52. 10.1038/s41398-020-01189-3 33446652 PMC7809257

[B37] Soccol C. R. Bissoqui L. Y. Rodrigues C. Rubel R. Sella S. R. Leifa F. (2016). Pharmacological properties of biocompounds from spores of the Lingzhi or reishi medicinal mushroom Ganoderma lucidum (agaricomycetes): a review. Int. J. Med. Mushrooms 18 (9), 757–767. 10.1615/IntJMedMushrooms.v18.i9.10 27910768

[B38] Souza L. C. Andrade M. K. Azevedo E. M. Ramos D. C. Bail E. L. Vital M. (2022). Andrographolide attenuates short-term spatial and recognition memory impairment and neuroinflammation induced by a streptozotocin rat model of Alzheimer's disease. Neurotox. Res. 40 (5), 1440–1454. 10.1007/s12640-022-00569-5 36029454

[B39] Swallah M. S. Bondzie-Quaye P. Wu Y. Acheampong A. Sossah F. L. Elsherbiny S. M. (2023). Therapeutic potential and nutritional significance of Ganoderma lucidum - a comprehensive review from 2010 to 2022. Food Funct. 14 (4), 1812–1838. 10.1039/d2fo01683d 36734035

[B40] Torterolo P. Sampogna S. Chase M. H. (2011). A restricted parabrachial pontine region is active during non-rapid eye movement sleep. Neuroscience 190, 184–193. 10.1016/j.neuroscience.2011.06.018 21704676 PMC3169006

[B41] Vicente M. C. Paneghini J. L. Stabile A. M. Amorim M. Anibal Silva C. E. Patrone L. G. A. (2023). Inhibition of pro-inflammatory microglia with minocycline improves cognitive and sleep-wake dysfunction under respiratory stress in a sporadic model for Alzheimer's disease. J. Alzheimers Dis. 95 (1), 317–337. 10.3233/JAD-230151 37522205

[B42] Yu N. Huang Y. Jiang Y. Zou L. Liu X. Liu S. (2020). Ganoderma lucidum triterpenoids (GLTs) reduce neuronal apoptosis via inhibition of ROCK signal pathway in APP/PS1 transgenic Alzheimer's disease mice. Oxid. Med. Cell Longev. 2020, 9894037. 10.1155/2020/9894037 32089787 PMC7008260

[B43] Zhao H. L. Cui S. Y. Qin Y. Liu Y. T. Cui X. Y. Hu X. (2021). Prophylactic effects of sporoderm-removed Ganoderma lucidum spores in a rat model of streptozotocin-induced sporadic Alzheimer's disease. J. Ethnopharmacol. 269, 113725. 10.1016/j.jep.2020.113725 33352241

[B44] Zheng G. Zhao Y. Li Z. Hua Y. Zhang J. Miao Y. (2023). GLSP and GLSP-derived triterpenes attenuate atherosclerosis and aortic calcification by stimulating ABCA1/G1-mediated macrophage cholesterol efflux and inactivating RUNX2-mediated VSMC osteogenesis. Theranostics 13 (4), 1325–1341. 10.7150/thno.80250 36923537 PMC10008734

